# The Decellularized Cell-Derived Extracellular Matrix Enhances the Paracrine Function of Human Mesenchymal Stromal/Stem Cells

**DOI:** 10.3390/ijms25042419

**Published:** 2024-02-19

**Authors:** Roman Ushakov, Andrey Ratushnyy, Ludmila Buravkova, Elena Tolkunova, Elena Burova

**Affiliations:** 1 Institute of Cytology, Russian Academy of Sciences, 194064 Saint Petersburg, Russia; uszakow@yandex.ru (R.U.); entolk62@mail.ru (E.T.); 2 Institute of Biomedical Problems, Russian Academy of Sciences, 123007 Moscow, Russia; ratushkin@mail.ru (A.R.); buravkova@imbp.ru (L.B.)

**Keywords:** mesenchymal stromal/stem cells (MSCs), decellularized extracellular matrix (dECM), cell preconditioning, secretome, paracrine factors, angiogenesis, focal adhesion kinase (FAK)

## Abstract

The mesenchymal stromal/stem cells (MSCs) are known to secrete pleiotropic paracrine factors, contributing to tissue regeneration. This unique ability makes MSCs promising therapeutic tools for many diseases, including even those that were previously untreatable. Thus, the development of preconditioning approaches aimed at enhancing the paracrine function of MSCs attracts great interest. In the present work, we studied how the extracellular matrix, the essential part of the native tissue microenvironment, affects the secretory capacity of MSCs of various origins. The MSC-derived decellularized extracellular matrix (dECM), used as the cell culture substrate, triggered strong upregulation of FGF-2, MMP-1, HGF, GRO-α, GRO-β, CXCL-5, CXCL-6, IL-6, IL-8, G-CSF and MCP-1. Functional in vitro tests revealed that conditioned media derived from MSCs cultured on dECM significantly improved 3T3 fibroblast and HaCaT keratinocyte scratch wound healing, stimulated THP-1 monocyte migration and promoted capillary-like HUVEC-based tube formation compared to conditioned media from MSCs grown on plastic. In addition, we found that FAK inhibition promoted dECM-induced upregulation of paracrine factors, suggesting that this kinase participates in the MSCs’ paracrine response to dECM. Together, these findings demonstrate that dECM provides cues that considerably enhance the secretory function of MSCs. Thus, dECM usage as a cell culture substrate alone or in combination with a FAK inhibitor may be viewed as a novel MSC preconditioning technique.

## 1. Introduction

The mesenchymal stromal/stem cells (MSCs) are multipotent adult stem cells that can be isolated from numerous tissues (bone marrow, adipose tissue, Wharton’s jelly of the umbilical cord, peripheral blood, periodontal ligaments, etc.) and expanded in vitro [1]. MSCs have been tested in hundreds of clinical trials as candidates for the treatment of various diseases because of the multiple biological activities they exert, including immunomodulatory, antimicrobial, anti-tumor, anti-apoptotic and antioxidative actions, stimulation of tissue regeneration and angiogenesis [2,3]. To a great extent, the therapeutic potential of MSCs is related to their secretome, which contains a plethora of soluble molecules (growth factors, cytokines, chaperones, proteinases, etc.) as well as the extracellular vesicles loaded with proteins and nucleic acids [4].

As the source of therapeutically beneficial molecules, MSCs can additionally be primed in order to boost their secretory function. Among the factors affecting the MSCs’ secretion, the following can be listed: pharmacological compounds, cytokines, a hypoxic atmosphere, 3D culture and substrate stiffness [5,6].

The extracellular matrix (ECM), the complex meshwork consisting of fibrillar glycoproteins, proteoglycans, associated proteins and deposited soluble factors, has a comprehensive impact on cell functions and provides the suitable microenvironment [7]. A decellularized ECM (dECM), i.e., subjected to the elimination of cell components by various physical and chemical methods, can be derived from tissues/organs or deposited in vitro by a cell culture, and it has been shown to be a more physiologically relevant substrate compared to the routinely used culture plastic by maintaining stem cells in an undifferentiated state, decelerating replicative senescence and decreasing intracellular levels of reactive oxygen species [8,9]. Regarding MSCs, dECM significantly stimulates their proliferation capacity, modulates differentiation potential and reduces the number of chromosomal abnormalities [10,11]. However, little is known about how the culture on dECM influences the MSCs’ secretory function. Nevertheless, there are several papers reporting the upregulation of some paracrine factors, such as IL-6, IL-8, MCP-1, HGF and GRO-α, in MSCs cultured on purified ECM proteins or in a hydrogel equipped with peptides targeting specific integrins [12,13,14].

The purpose of our study was to place MSCs in a more suitable microenvironmental context provided by dECM in an effort to enhance their secretory function and to improve their therapeutic potential. Based on the assumption that dECM, as the depot of ligands for integrin receptors, supplies multiple signals that a cell is deprived of while being maintained on standard culture plastic, we hypothesized the modulating role of dECM in the MSCs’ secretory activity. In the present work, we studied how dECM affects the expression of paracrine factors in MSCs of various origins and which functionalities the conditioned medium acquires upon MSC culture on dECM. Using the inhibitory analysis, we also attempted to identify the potential regulatory molecules involved in the transduction of dECM signals.

## 2. Results

### 2.1. Production and Characterization of Bioactive dECM

**Figure 1:**
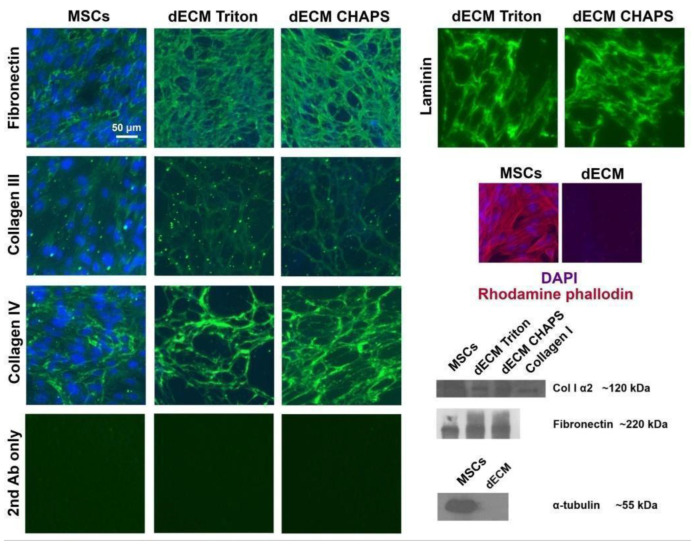
Immunofluorescence and immunoblotting images of the extracellular matrix prior to decellularization (MSCs) and after (dECM Triton and dECM CHAPS according to decellularization method, respectively). Staining with secondary antibodies only was used as the autofluorescence control.

**Figure 2:**
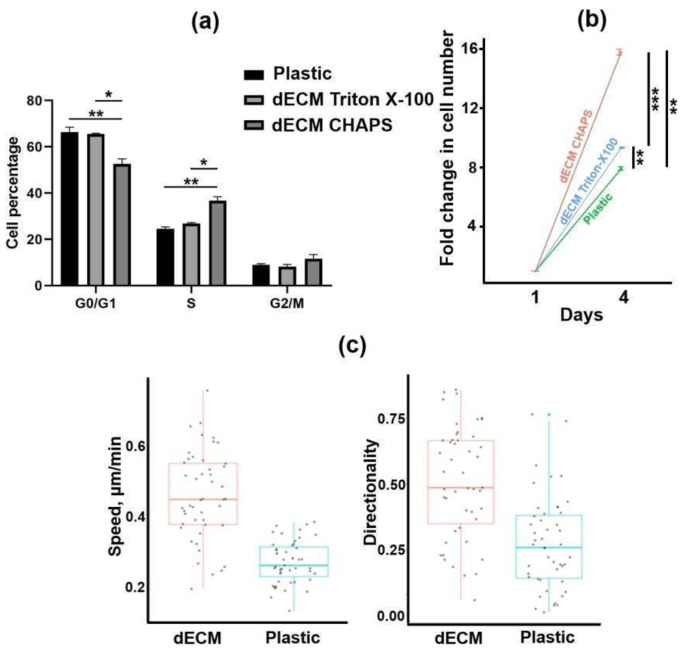
Characterization of dECM biological activity. (**a**) Cell cycle analysis of MESCs cultured on plastic, Triton-treated dECM and CHAPS-treated dECM performed with the use of flow cytometry; (**b**) Fold change in the total number of cells grown on different surfaces by the 4th day. (**c**) Speed and directionality of motion of MESCs seeded on CHAPS-treated dECM and plastic; data are calculated on the basis of individual cell tracks obtained with the use of confocal imaging cytometer Yokogawa CQ1; values are presented as boxplots with minimum, maximum, mean, 25th and 75th percentile (n = 50). In panels (**a**,**b**), values are presented as means ± SD (n = 3). *—*p* < 0.05; **—*p* < 0.01; ***—*p* < 0.001 (ANOVA with post hoc Tukey’s test).

First, we optimized the production of dECM with retention of its biological activity. Wharton’s jelly-derived MSCs were chosen as the ECM producers. We tested two commonly used methods for decellularization of MSC-deposited ECM: 0.5% Triton X-100 and 0.5% 3-[(3-cholamidopropyl) dimethylammonio]-1-propanesulfonate (CHAPS) detergents, both alkalinized with 20 mM ammonium hydroxide. Both methods demonstrated efficient elimination of cellular nuclei, actin and tubulin cytoskeletons with preservation of major ECM components, such as collagen types I, III and IV, fibronectin and laminin (Figure 1). Next, we tested whether Triton- and CHAPS-treated matrices promote the proliferation of human endometrium-derived MSCs (MESCs). Surprisingly, compared to culture plastic, only the CHAPS-treated ECM significantly increased the proportion of cells in the S phase with a substantial increment in total cell number (Figure 2a,b). Scanning electron microscopy revealed that Triton-treated ECM fibrils are covered with some particles that could prevent the interaction of cell receptors with ECM (Figure S1). In addition, using time-lapse live-cell imaging, we found that the MESCs seeded on CHAPS-treated ECM migrated with a significantly higher speed and directionality score compared to the MESCs cultured on plastic (Figure 2c). Thus, we considered CHAPS-treated dECM as bioactive and used CHAPS-based decellularization in further work.

### 2.2. Expression of Paracrine Factors by MSCs Cultured on dECM

**Figure 3:**
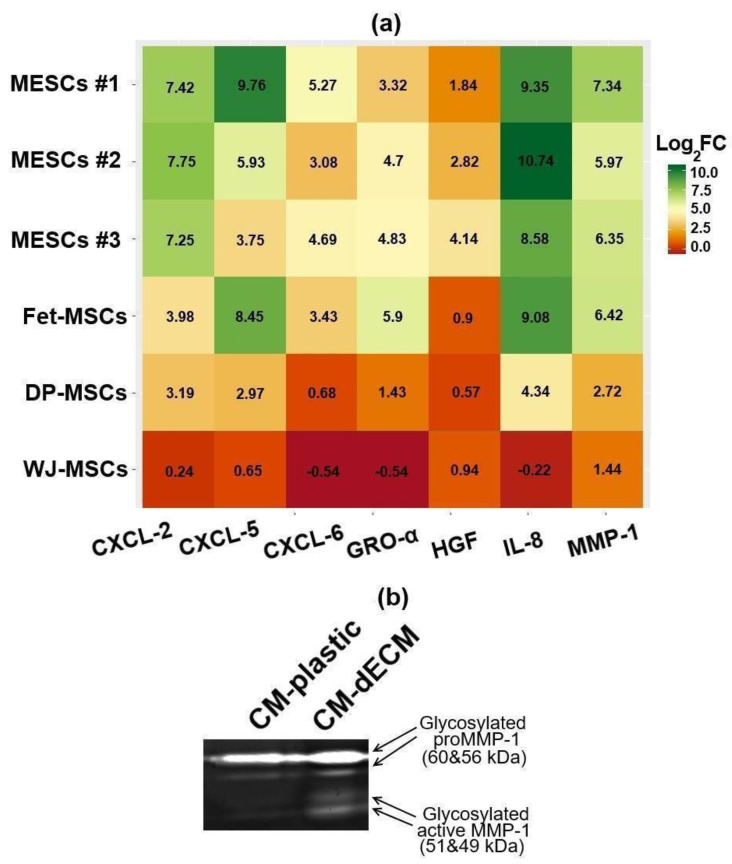
Culture on dECM within 72 h affected expression of paracrine factors by MSCs. (**a**) RT-qPCR heatmap depicts differential expression (dECM culture vs. plastic culture) of paracrine factor mRNA in MSCs of various origins. Values are presented as means (n = 3) of binary logarithms of mRNA expression fold change. GAPDH was used as a reference gene. MESCs—human endometrium-derived MSCs (obtained from three donors), Fet-MSCs—human fetal bone marrow-derived MSCs, DP-MSCs—dental pulp-derived MSCs, WJ-MSCs—Wharton’s jelly-derived MSCs; (**b**) MMP-1 activity (collagen zymography) in conditioned media of MESCs cultured on plastic (CM-plastic) and dECM (CM-dECM).

We used RT-qPCR in order to test how dECM affects the expression of some genes coding paracrine factors in MESCs, fetal bone marrow-derived MSCs (Fet-MSCs), dental pulp-derived MSCs (DP-MSCs) and Wharton’s jelly-derived MSCs (WJ-MSCs). As shown in Figure 3a, dECM triggers the upregulation of matrix metalloproteinase 1 (MMP-1), hepatocyte growth factor (HGF) and the C-X-C motif ligand (CXCL) family chemokines GRO-α (CXCL-1), GRO-β (CXCL-2), CXCL-5, CXCL-6 and IL-8 (CXCL-8). Interestingly, MSCs of different origins varied in upregulation levels: the strongest upregulation was observed (up to three orders of magnitude for IL-8 and up to two orders for MMP-1 and CXCLs) in MESCs and Fet-MSCs, while DP-MSCs and WJ-MSCs demonstrated the weakest upregulation. Notably, MESCs cultured on the dECMs produced by the MSCs of three various sources displayed similar upregulation patterns (Figure S2). MMP-1 upregulation in conditioned medium of MESCs cultured on dECM was confirmed by means of collagen zymography (Figure 3b). Next, we performed a multiplex bead-based immunoassay to detect the levels of cytokines, chemokines and growth factors in conditioned media of MSCs cultured on both plastic and dECM. Similar to the RT-qPCR analysis, the multiplex immunoassay showed dECM-induced upregulation of HGF, GRO-α and IL-8 in all studied MSCs and additionally revealed upregulation of fibroblast growth factor (FGF-2), granulocyte colony-stimulating factor (G-CSF) and chemokines IL-6 and MCP-1 at least in three kinds of studied MSCs (Table 1). Thus, dECM triggered considerable upregulation of a number of paracrine factors in MSCs.

### 2.3. Conditioned Medium of MESCs Cultured on dECM Acquired Chemotactic and Angiogenic Properties

**Figure 4:**
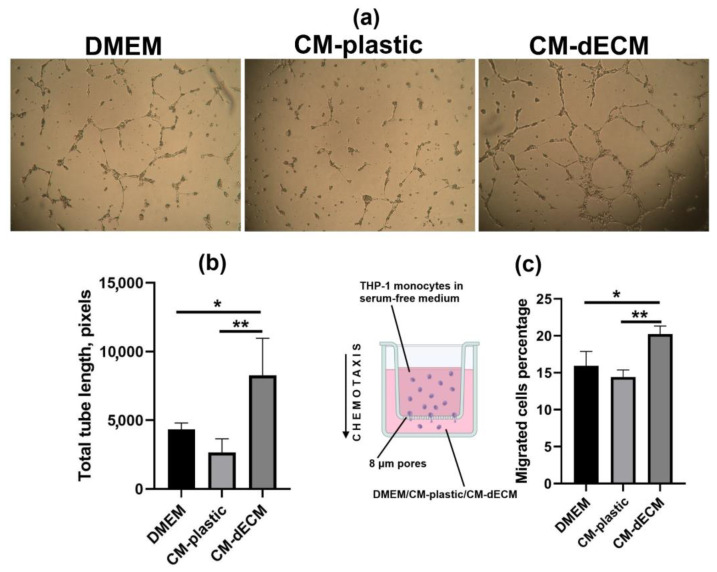
Conditioned medium of MESCs cultured on dECM acquired angiogenic and chemotactic properties. (**a**) Representative image of the human umbilical vein endothelial cells (HUVECs) tube formation on basal membrane extract taken at 24 h after the addition of conditioned media; (**b**) Tube length quantification, *—*p* < 0.05, **—*p* < 0.01 (ANOVA with post hoc Tukey’s test); (**c**) THP-1 transwell migration assay *—*p* < 0.05; **—*p* < 0.01 (ANOVA with post hoc Tukey’s test). In panels (**b**,**c**), values are presented as means ± SD (n = 3).

Taking into account the significant upregulation of several CXCL family members known for inducing leukocyte migration to inflammatory sites, we additionally validated the upregulation of chemokines by assessing THP-1 monocyte migration through the 8 μm pores of a transwell insert polycarbonate membrane towards the lower chamber filled with serum-free DMEM-F/12 or conditioned media of MESCs cultured either on plastic (CM-plastic) or on dECM (CM-dECM). At 24 h after the beginning of the assay, there were significantly more THP-1 cells that migrated towards CM-dECM than to CM-plastic and DMEM-F/12, indicating that CM-dECM contained more chemoattractants (Figure 4c).

Considering the upregulation of paracrine factors exerting angiogenic action, such as IL-8, GRO-α, HGF and FGF-2, we used the tube formation assay in order to test whether CM-dECM promotes angiogenesis. Human umbilical vein endothelial cells (HUVECs) were seeded on a basement membrane extract-based gel with the addition of DMEM/F-12, CM-plastic or CM-dECM. Tweny-four hours later, the HUVECs cultured in the presence of CM-dECM formed significantly more capillary-like structures compared to the ones cultured with DMEM/F-12 and CM-plastic (Figure 4a,b). Thus, the chemotaxis and angiogenesis assays revealed functional changes in the secretome of MESCs cultured on dECM.

### 2.4. Conditioned Media of MSCs Cultured on dECM Stimulated 3T3 Fibroblast and HaCaT Keratinocyte Scratch Wound Healing

**Figure 5:**
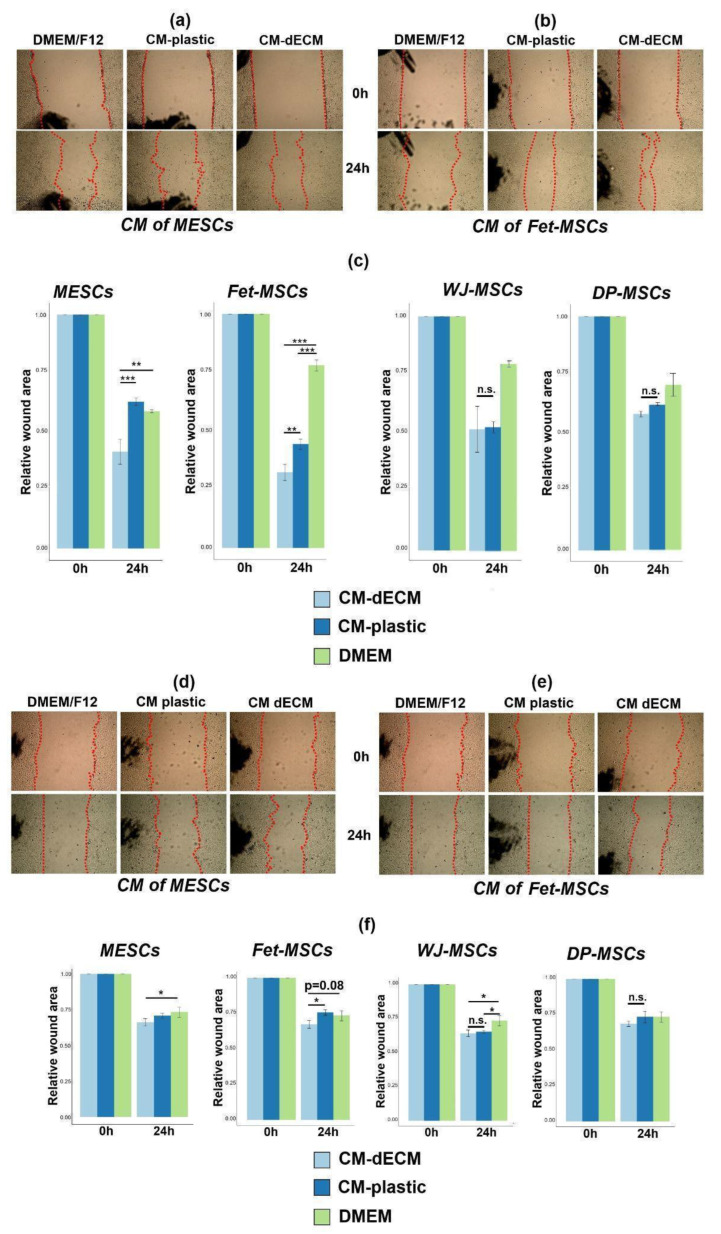
Conditioned medium of MSCs cultured on dECM stimulated 3T3 fibroblast and HaCaT keratinocyte scratch wound healing. (**a**,**b**) Representative images depicting 3T3 wound healing with conditioned media of MESCs (**a**) and Fet-MSCs (**b**); (**c**) Quantification of 3T3 scratch wound assay, *—*p* < 0.05, **—*p* < 0.01, ***—*p* < 0.001 (ANOVA with post hoc Tukey’s test); (**d**,**e**) Representative images depicting HaCaT wound healing with conditioned media of MESCs (**d**) and Fet-MSCs (**e**); (**f**) Quantification of HaCaT scratch wound assay, *—*p* < 0.05, **—*p* < 0.01 (ANOVA with post hoc Tukey’s test). In panels (**c**,**f**), values are presented as means ± SD (n = 3). Red line in panels (**a**,**b**,**d**,**e**) indicates margins of scratch wounds.

We took into consideration that upregulated paracrine factors are reported to possess therapeutic potential, and we tested the properties of conditioned media in scratch assays using 3T3 fibroblasts and HaCaT keratinocytes as the in vitro models of wound healing. We found that CM-dECM of both MESCs and Fet-MSCs promoted closure of 3T3 and HaCaT wounds compared to DMEM/F-12 and CM-plastic, while CM-dECM of DP-MSCs and WJ-MSCs did not (Figure 5). Notably, these differences in dECM-induced scratch wound healing stimulation between MSCs of various origins correlated with differences in the degree of dECM-triggered upregulation of paracrine factors (Figure 3a and Table 1). In addition, we revealed that dECM significantly contributed to the proliferation of MESCs and Fet-MSCs but not of DP-MSCs and WJ-MSCs (Table S1). Apparently, MSCs of various origins may have different receptivity to signals from dECM, and in addition, donor-to-donor variations should also be considered. Together, our findings suggest that dECM is capable of triggering changes in conditioned media of MESCs and Fet-MSCs that stimulate in vitro wound healing. Given that CM-dECM is enriched in chemokines and growth factors, it can be hypothesized that improved scratch wound healing may occur due to both enhanced migration and proliferation of 3T3 and HaCaT.

### 2.5. FAK Inhibition Promoted dECM-Induced Upregulation of Paracrine Factors

**Figure 6:**
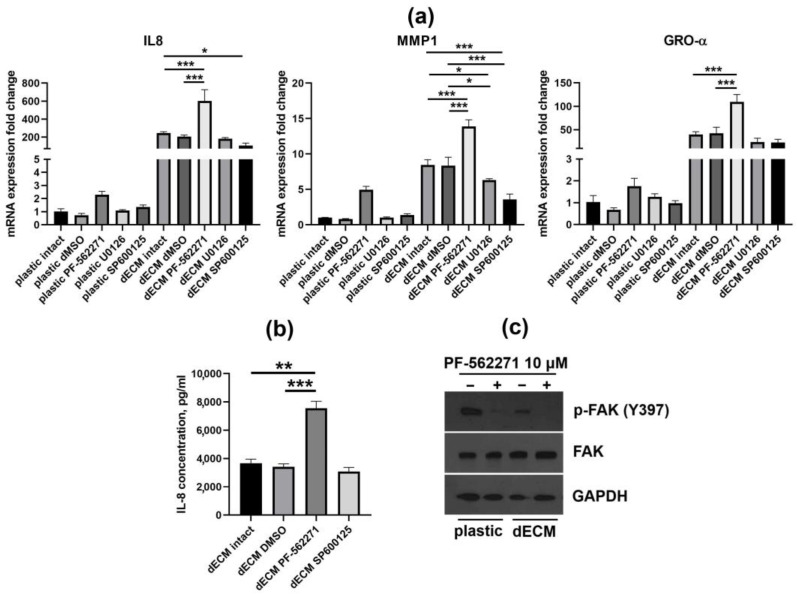
Inhibitory analysis of signal pathways related to the dECM-induced paracrine factor upregulation in MESCs. (**a**) RT-qPCR estimation of IL-8, MMP-1 and GRO-α mRNA expression in MESCs cultured within 24 h upon treatment with the following inhibitors: 10 μM PF-562271 (FAK inhibitor), 10 μM U0126 (MEK1/2 inhibitor) and 5 μM SP600125 (JNK inhibitor); (**b**) ELISA estimation of IL-8 concentration in conditioned media of MESCs cultured on dECM within 48 h upon treatment with FAK and JNK inhibitors. IL-8 level in conditioned media of MESCs cultured on plastic was undetectable; (**c**) FAK phosphorylation (Y397) status in MESCs cultured on plastic and dECM within 72 h and treated with FAK inhibitor. In panels (**a**,**b**), values are presented as means ± SD (n = 3). *—*p* < 0.05, **—*p* < 0.01, ***—*p* < 0.001 (ANOVA with post hoc Tukey’s test). Treatment with DMSO was used as vehicle control.

Next, we applied an inhibitory analysis in order to estimate which signal pathways may be involved in dECM-induced paracrine factor upregulation. The focal adhesion kinase (FAK), the key component of intracellular cascades transmitting signals from dECM, was chosen as an inhibition target. In addition, since MSCs of various origins demonstrated correlation between dECM-triggered paracrine response and dECM-induced stimulation of MSC proliferation, we also considered the possible participation of mitogen-activated protein kinase (MAPK) pathways, in particular, JNK and MEK1/2. We performed RT-qPCR analysis of IL-8, MMP-1 and GRO-α mRNA expression in MESCs cultured on plastic and dECM upon treatment with inhibitors of FAK (PF-562271), JNK (SP600125) and MEK1/2 (U0126) (Figure 6a). Inhibition of FAK significantly enhanced (approximately two- to three-fold) the dECM-triggered upregulation of IL-8, MMP-1 and GRO-α. Of note, in cells cultured on dECM, we observed a notable decrease in FAK phosphorylation (Y397) even without treatment with a FAK inhibitor (Figure 6c), suggesting that FAK may serve as a negative regulator of paracrine factor expression. Conversely, JNK inhibition led to a statistically significant decrease in both IL-8 (compared to intact untreated cells but not to DMSO vehicle control) and MMP-1 levels in MESCs cultured on dECM, while MEK inhibition diminished only MMP-1 expression. Both JNK and MEK inhibitors slightly decreased the GRO-α level. An enzyme-linked immunosorbent assay (ELISA) assay revealed a considerable increase in IL-8 concentration in conditioned medium of the FAK inhibitor-treated MESCs cultured on dECM consistently with the RT-qPCR results. At the same time, JNK inhibition had no noticeable effect on IL-8 concentration (Figure 6b). Thus, we conclude that, unlike JNK and MEK inhibition, FAK inhibition has a pronounced positive effect on dECM-triggered paracrine factor upregulation.

## 3. Discussion

In the present work, we showed that the culture on cell-derived dECM led to the manifold upregulation of a number of paracrine factors in MSCs, such as FGF-2, MMP-1, HGF, GRO-α, GRO-β, CXCL-5, CXCL-6, IL-6, IL-8, G-CSF and MCP-1. In addition, several in vitro assays (monocyte chemotaxis, capillary-like tube formation and fibroblast/keratinocyte scratch wound healing) provided evidence of functional changes in the MSCs’ secretome upon culture on dECM. Many upregulated factors have been reported to contribute to regeneration in various tissues. For instance, Choi et al. demonstrated that IL-8 secreted by adipose tissue-derived MSC (AT-MSC) spheroids promoted angiogenesis and muscle regeneration in the ischemic murine hindlimb [15]. Likewise, IL-8 was shown to stimulate osteochondral regeneration in a beagle model [16]. MMP-1 treatment accelerated wound closure, improved angiogenesis and revascularization, improved nerve regeneration and reduced fibrosis formation in amputated murine digits [17]. Several papers reported that HGF secreted by genetically modified MSCs showed a therapeutic effect in murine models of myocardial infarction, radiation-induced intestinal injury and liver fibrosis [18,19,20,21]. G-CSF was shown to exert pleiotropic actions by stimulating bone regeneration and improving outcomes in animal models of neurological and cardiovascular diseases [22,23]. FGF-2 promoted the healing of skin wounds, diabetic ulcers, spinal cord damage and bone fractures [24]. In this regard, the use of dECM as a culture substrate or as a component of bioengineered scaffolds may be beneficial for cell-based therapies. However, the effects of dECM on MSC paracrine function should be further validated with in vivo experiments in order to evaluate the relevance of this preconditioning approach. Our future efforts will focus on testing the CM-dECM properties on animal models.

Remarkably, our results are consistent with the findings of Ragelle et al. who performed a transcriptomic analysis of bone marrow-derived MSCs (BM-MSCs), AT-MSCs and neonatal human dermal fibroblasts (NHDF) subjected to culture on dECMs deposited by each of these cell types (nine combinations in total) [25]. A microarray assay revealed a similar upregulation pattern with an increase in MMP-1, IL-8, GRO-α, CXCL-2, CXCL-5 and CXCL-6 mRNA levels in BM-MSCs and AT-MSCs but not in NHDF (Figure S3). Interestingly, the cell responses to each dECM type were comparable, suggesting that some intrinsic cellular properties rather than dECM composition determine the cells’ ability to upregulate paracrine factors upon exposure to dECM. Analyzing the transcriptomics data published by Ragelle et al., we noticed that, in comparison to NHDF, BM-MSCs and AT-MSCs had significantly different profiles of integrin subunit expression (Figure S4), and this provides a possible explanation of why cells vary in their responses to dECM. In our study, MSCs of different origins also varied in the magnitude of the paracrine response to dECM.

Kim et al. found that cytokine, chemokine and growth factor genes, including IL-6, GRO-α, CXCL-2, CXCL-5, CXCL-6, IL-8 and MCP-1, were upregulated in AT-MSCs cultured in high density for 7 days [26]. Considering an upregulation pattern that is very similar to that studied in our work, it could be proposed that the AT-MSCs received signals from the ECM that affected the expression of the mentioned genes because 7 days may be sufficient to form ECM by a cell culture in vitro. Peng et al. showed that placenta-derived MSCs cultured on plates coated with ECM protein laminin enhanced secretion of IL-8, GRO-α and HGF, and this effect was mediated through the αvβ3 integrin receptor [13]. Clark et al. produced hydrogels modified with GFOGER and RGD peptides having high affinity to different integrin heterodimers and detected significant upregulation of IL-6, IL-8, IL-9, MCP-1 and VEGF in BM-MSCs cultured on these hydrogels [14]. Together, these works support the suggestion that regulators of paracrine factor expression in MSCs may be sensitive to signals from dECM. Interestingly, Alexandrushkina et al. found application of cell sheets from AT-MSCs to be more efficient in the healing of pressure ulcers than injection of suspended MSCs, and this effect was linked to the enhanced paracrine function of the cell sheet, producing more HGF, G-CSF, PDGF-BB and Ang-2 [27]. It should be noticed that ECM proteins are abundant in a cell sheet, so ECM’s role in modulating the secretory function of AT-MSCs within the cell sheet may be proposed.

The focal adhesion kinase (FAK) is a tyrosine kinase that mediates the transduction of signals from integrin receptors to the downstream targets, such as PI3K/Akt and MAPKs [28]. FAK is activated by autophosphorylation at Y397 upon integrin binding to ECM proteins and to other ligands [28]. In the present study we found that dECM-triggered paracrine factor upregulation coincided with a decrease in the level of FAK phosphorylation at Y397. Further inhibition of FAK phosphorylation with the PF-562271 inhibitor increased the expression of IL-8, MMP-1 and GRO-α. This allows for the suggestion that FAK plays the role of a negative regulator of paracrine factor expression in MSCs. Thus, a combination of culture on dECM with FAK inhibition synergistically enhanced MSC paracrine function, so it may be considered as a novel MSC priming approach. However, it should be kept in mind that a kinase inhibitor may exert off-target effects that are difficult to distinguish from on-target effects, so the potential FAK involvement in the MSC paracrine response to dECM should be further corroborated with a more specific method such as gene knockdown. Although we did not find any works elucidating how FAK inhibition affects the paracrine function of MSCs, a similar phenomenon was observed in RAW 264.7 macrophages that underwent substantial upregulation of a number of cytokines and chemokines (up to a hundred-fold increase) upon treatment with a FAK inhibitor [29]. MCF-7 breast cancer cells cultured for 24 h on dECM [30] as well as AT-MSCs (culture period was not indicated) [10] were reported to have increased levels of Y397-phosphorylated FAK, while we observed an inverse effect in MESCs cultured on dECM for 72 h. This inconsistency needs to be further investigated by evaluating the dynamics of FAK phosphorylation in MSCs exposed to dECM.

Discussing the limitations of our study, constraints of MSC sample size should be stressed. We used an MSC sample that can only reflect the general trend of paracrine factor upregulation in MSCs exposed to dECM. Because of a lack of biological repeats for MSCs of various origin, it is not clear whether MSCs differ in ability to dECM-induced paracrine response due to donor-to-donor variations or due to some properties related to their origin. We also cannot propose dECM as the universal culture substrate that improves the secretory activity of any MSCs due to the lack of such information. In addition, it is merely possible to control dECM production by cell culture since it is a complex structure composed of dozens of components. So, the implementation of the dECM culture substrate into large-scale expansion of MSCs or manufacturing of MSC-derived products would face the problem of standardization in the preparation of dECM. Even commercially available ECM extracts such as Matrigel are known to have batch-to-batch variations. However, in order to maintain result consistency in our study, we strictly observed the usage of only one batch of both culture medium with supplements and decellularization reagent. Moreover, for dECM preparation, we used cells from the same frozen batch of only one donor. To ensure reproducibility of dECM functionalities, we also tested every new dECM batch with RT-qPCR for paracrine factor expression and with a proliferation assay.

In conclusion, our study showed that cell-derived dECM, a physiologically relevant culture substrate, boosted the production of a number of soluble paracrine factors in MSCs of various origins. dECM-induced upregulation improved the angiogenic and chemotactic properties of MSC-derived conditioned medium and stimulated scratch wound healing. In addition, treatment with a FAK inhibitor resulted in the enhancement of dECM-triggered paracrine factor upregulation. These findings provide novel ideas on how to improve the efficiency of MSC-based therapies.

## 4. Materials and Methods

### 4.1. Cells

MESCs, Fet-MSCs, WJ-MSCs, DP-MSCs, 3T3 and THP-1 cells were obtained from the shared research facility Vertebrate Cell Culture Collection of the Institute of Cytology, RAS. All studied MSCs were previously shown to express CD73, CD90 and CD105, to be negative for CD34 and CD45 surface markers and to have the ability to differentiate into osteoblasts, chondrocytes and adipocytes [31,32,33,34]. MSCs were cultured in DMEM/F12 growth medium (Gibco, Grand Island, NY, USA) supplemented with 10% fetal bovine serum (FBS, HyClone, Pasching, Austria), 1% penicillin–streptomycin (Biolot, Saint Petersburg, Russia) and 1% GlutaMax (Gibco, Grand Island, NY, USA) at +37 °C in a humidified incubator with 5% CO_2_. Cells were subcultured 1:3 every 72 h with 0.05% EDTA–trypsin solution (Gibco, Grand Island, NY, USA) within 6–12 passages. The 3T3 cultivation conditions were the same as for the MSCs. THP-1 cells were cultivated in RPMI-1640 growth medium (Biolot, Saint-Petersburg, Russia) supplemented with 10% FBS, 1% penicillin–streptomycin and 1% GlutaMax at +37 °C in a humidified incubator with 5% CO_2_ and subcultured 1:5 every 72 h.

HaCaT cells were obtained from the shared research facility Cell Culture Collection of the Koltsov Institute of Developmental Biology, RAS. HaCaT cells were cultured in DMEM High Glucose supplemented with 5% FBS, 1% penicillin–streptomycin and 1% GlutaMax at +37 °C in a humidified incubator with 5% CO_2_ and subcultured 1:5 with 0.05% EDTA–trypsin every 72 h.

HUVECs were obtained from the Almazov National Medical Research Center of the Ministry of Health of Russia. HUVECs were cultured in EGM2 basal medium (PromoCell, Heidelberg, Germany) supplemented with 2% FBS (PromoCell, Heidelberg, Germany), 1% penicillin–streptomycin (Gibco, Grand Island, NY, USA) and hEGF 5 ng/mL, hydrocortisone-100 0.2 μg/mL, hVEGF 0.5 ng/mL, hgFGF 10 ng/mL, R3 IGF 22 ng/mL, ascorbic acid-500 1 μg/mL and heparin 22.5 ng/mL (Supplement Pack, PromoCell, Heidelberg, Germany). Cells were subcultured once weekly with Accutase (Gibco, Grand Island, NY, USA) up to passage 6.

### 4.2. Preparation of dECM

A total of 2 × 10^5^ WJ-MSCs were seeded into polystyrene Nunclon™ Delta 35 mm dishes and 6-well plates (Thermo Fisher Scientific, Waltham, MA, USA) that were precoated with 0.1% bovine skin gelatin (Sigma-Aldrich, St. Louis, MO, USA) for 30 min at +37 °C. Every 72 h, complete growth medium was changed to fresh with the addition of 50 μg/mL 2-phospho-L-ascorbic acid trisodium salt (Sigma-Aldrich, St. Louis, MO, USA). Fourteen days later, the dishes were washed twice with PBS and then treated with 1 mL of 0.5% CHAPS (Sigma-Aldrich, St. Louis, MO, USA) + 20 mM ammonium hydroxide in PBS solution for 3 min and carefully washed with PBS three times. During the washes, all solutions were discarded without use of the pipette in order not to disturb the fragile dECM layer. Dishes with damaged dECM were rejected and not further used. dECM-coated dishes were stored at +4 °C filled with PBS + 1% penicillin–streptomycin within a month until further use.

### 4.3. Immunofluorescence

dECM samples were prepared on coverslips as described above and then fixed with 3.7% formalin (Sigma-Aldrich, St. Louis, MO, USA) for 15 min, permeabilized with 0.5% Triton-X100 (Sigma-Aldrich, St. Louis, MO, USA) for 15 min, blocked with 1% bovine serum albumin (BSA, Sigma-Aldrich, St. Louis, MO, USA) and stained overnight at +4 °C with the following primary antibodies: anti-fibronectin (number F3648, Sigma-Aldrich, St. Louis, MO, USA), anti-collagen type III (number RAH C33) and anti-collagen type IV (number RAH C44, both from Imtek, Moscow, Russia), and anti-laminin (number ab11575, Abcam, Cambridge, UK). Next, the coverslips were washed with PBS three times for 15 min, stained with secondary Alexa Fluor™ 488-conjugated goat anti-rabbit antibodies (number A-11008, Thermo Fisher Scientific, Waltham, MA, USA) and mounted with DAPI/Antifade Solution (Sigma-Aldrich, St. Louis, MO, USA). Antibodies were diluted according to the recommendations of the manufacturers. Observation of the stained samples and image acquisition were performed with the use of the ZOE Fluorescent Cell Imager (Bio-Rad Laboratories Inc., Hercules, CA, USA).

### 4.4. Immunoblotting

SDS-PAGE and Western blotting were performed as described previously [35]. The Triton-insoluble fraction of the dECM lysate was dissolved in 8 M urea and mixed with the Triton-soluble fraction. The following primary antibodies were used: anti-collagen type I α2 chain (number SAB4500363), anti-fibronectin (number F3648) and anti-α-tubulin (number T5168, all from Sigma-Aldrich, St. Louis, MO, USA); anti-phopsho-FAK(Y397) (number 3283S), anti-FAK (number 3285S) and anti-GAPDH (number 2118S, all from Cell Signaling, Danvers, MA, USA). Antibodies were diluted according to the recommendations of the manufacturers.

### 4.5. Analysis of MESC Proliferation on dECM

A total of 3 × 10^4^ MESCs were seeded into 35 mm polystyrene and dECM-coated dishes. Twenty-four hours later, the cells were trypsinized and concentrated by centrifugation. Then, the cells were permeabilized with 0.1% Triton-X100 and stained for 5 min with 2 μg/mL DAPI at room temperature. Cell cycle phase distribution was revealed with use of a CytoFlex flow cytometer (Beckman Coulter, Fullerton, CA, USA) and CytExpert 2.0 software (Beckman Coulter, Fullerton, CA, USA). Ninety-six hours after seeding, the number of grown cells was measured with the use of flow cytometry.

### 4.6. Analysis of MESC Migration on dECM

A total of 5 × 10^4^ MESCs were seeded into the wells of a 6-well plate (polystyrene and dECM-coated) in complete growth medium with the addition of 1 μg/mL Hoechst 33,342 fluorescent dye (Thermo Fisher Scientific, Waltham, MA, USA). Then, the plate was cultivated for 24 h at 37 °C and 5% CO_2_ in the CQ1 Confocal Imaging Cytometer (Yokogawa Electric Corp., Tokyo, Japan). Twenty fields of view were photographed every 15 min. Time-lapse series of merged phase-contrast and fluorescent images were analyzed with Manual Tracking ImageJ plugin in order to obtain individual tracks of 50 cells. Mean cell speed and directionality score were calculated using Chemotaxis and Migration Tool software Version 2.0 (Ibidi GmbH, Martinsried, Germany).

### 4.7. RT-qPCR

RNA was isolated using an RNeasy mini kit (Qiagen, Hilden, Germany) and quantified with Implen^™^ NanoPhotometer^™^ NP80 (Implen, Munich, Germany). cDNA was synthesized by means of the MMLV RT kit (Evrogen, Moscow, Russia) and amplified with specific primers (Beagle, Saint Petersburg, Russia) using 5× qPCRmix-HS SYBR (Evrogen, Moscow, Russia) in the CFX96 Touch Real-Time PCR Detection System (Bio-Rad Laboratories Inc., Hercules, CA, USA). Fold change in gene expression was calculated according to the Pfaffl method [36]. Expression of target genes was normalized to GAPDH expression. Primer sequences were as follows:

HGF F: 5′-GAGAGTTGGGTTCTTACTGCACG-3′

HGF R: 5′-CTCATCTCCTCTTCCGTGGACA-3′

MMP-1 F: 5′-ACAGCTTCCCAGCGACTCTA-3′

MMP-1 R: 5′-TTGCCTCCCATCATTCTTCAGG-3′

GRO-α F: 5′-ATATTTCTGAGGAGCCTGCAACA-3′

GRO-α R: 5′-CCCCTGCCTTCACAATGATCT-3′

CXCL-2 F: 5′-GGCAGAAAGCTTGTCTCAACCC-3′

CXCL-2 R: 5′-CTCCTTCAGGAACAGCCACCAA-3′

CXCL-5 F: 5′-CCAATCTTCGCTCCTCCAATCT-3′

CXCL-5 R: 5′-TGGACAGGAGGCTCATAGTGG-3′

CXCL-6 F: 5′-GAGATCCCTGGACCCAGTAAGA-3′

CXCL-6 R: 5′-GTAGGCTTTCCCCCACACTC-3′

IL-8 F: 5′-CCACCGGAAGGAACCATCTC-3′

IL-8 R: 5′-CAGGAAGGCTGCCAAGAGAG-3′

GAPDH F: 5′-GCCGCATCTTCTTTTGCGTC-3′

GAPDH R: 5′-AATCCGTTGACTCCGACCTTC-3′

### 4.8. Conditioned Medium Collection

MSCs were seeded on 35 mm plastic and dECM-coated dishes. Upon reaching 90% confluency, the MSCs were washed 5 times with PBS and then cultured for 48 h in serum-free DMEM/F12 supplemented with 1% penicillin–streptomycin and 1% GlutaMax. Conditioned medium was then collected, centrifuged at 2500× *g* to remove cell debris, equalized with the Bradford assay and stored at −80 °C until further use.

### 4.9. Multiplex Immunoassay

The concentrations of chemokines, cytokines and growth factors in the conditioned media were measured by means of the Milliplex^®^ MAP System using a Human Cytokine/Chemokine Magnetic Bead Panel (Merck Millipore, Darmstadt, Germany) according to the manufacturer’s protocol as described previously [37]. The concentrations of HGF were estimated with the use of the Bio-Plex Pro^™^ Human Cytokine Screening Panel (Bio-Rad Laboratories Inc., Hercules, CA, USA) according to the manufacturer’s instructions.

### 4.10. Collagen Zymography

MMP-1 activity in the conditioned media was evaluated as described by Inanc et al. [38]. A 10% SDS-PAGE gel was copolymerized with 0.3 mg/mL collagen type I extracted from rat tail tendons. Conditioned media samples were 10-fold concentrated with Pierce^™^ Protein Concentrators 3K MWCO (Thermo Fisher Scientific, Waltham, MA, USA), equalized with use of the Bradford assay, mixed with 5× non-reducing sample buffer (4% SDS, 20% glycerol, 0.01% bromophenol blue, 125 mM Tris-HCl pH 6.8) and electrophoresed. Then, the gel was washed 3 times for 15 min in 2.5% Triton-X100 and incubated in development buffer at +37 °C for 48 h with agitation (50 mM Tris-HCl pH 7.5, 10 mM CaCl_2_, 50 mM NaCl, 0.02% NaN_3_). Next, the gel was stained with 0.5% Coomassie Blue R-250 and destained with 40% methanol and 10% acetic acid solution, followed by scanning in the Chemidoc MP Imaging System (Bio-Rad Laboratories Inc., Hercules, CA, USA).

### 4.11. Scratch Wound Healing Assay

A total of 2 × 10^5^ HaCaT and 1.5 × 10^5^ 3T3 cells were seeded into 24-well plates and cultured until confluency. Then, the monolayers were scratched with a 200 μL pipette tip and washed with PBS three times, followed by adding 500 μL of studied conditioned media or control DMEM/F12. Only wells with a uniform initial scratch size were used in the assay in order to provide the correct normalization. The wounded monolayers were observed under a phase contrast inverted microscope (Nikon Eclipse TS100, Tokyo, Japan) and photographed (Canon EOS1000, Tokyo, Japan) immediately and 24 h after scratching. Areas of wound closure were determined manually by means of ImageJ Version 1.8.0 software, and relative wound areas were calculated as (wound area at 24 h)/(initial wound area) ratio.

### 4.12. Transwell Migration Assay

Twenty-four hours prior to the experiments, THP-1 cells were starved for serum. A total of 30 × 10^4^ THP-1 cells in 300 μL of serum-free RPMI-1640 were placed into 24-well, 8 μm transwell inserts (Kirgen Bioscience, Shanghai, China), while the lower chambers were filled with 500 μL of studied conditioned media or control DMEM/F12. Twenty-four hours later, the number of cells that migrated towards the lower chamber was estimated with the use of a flow cytometer.

### 4.13. Tube Formation Assay

The wells of a 96-well plate were coated with 50 μL of the pre-chilled 8–11 mg/mL Low-factor Star Matrigengel (ABW, Shanghai, China) on ice with pre-chilled pipette tips in order to prevent premature gelation and incubated overnight at +37 °C. Then, 3 × 10^4^ HUVECs in 50 μL of EGM basal medium were placed into the Matrigengel-coated wells. Next, 50 μL of studied conditioned media or control DMEM/F12 was added to the wells, followed by incubation at +37 °C and 5% CO_2_. Twenty-four hours later, the wells were photographed. The total lengths of the formed capillary-like tubes were measured using the ImageJ angiogenesis analyzer (http://image.bio.methods.free.fr/ImageJ/?Angiogenesis-Analyzer-for-ImageJ&artpage=2-6&lang=en, accessed on 28 November 2023).

### 4.14. IL-8 ELISA

The concentrations of IL-8 in the conditioned media were measured with the use of an IL-8 ELISA kit (Vektor-Best, Novosibirsk, Russia) according to the manufacturer’s instructions. Absorbance was read on the Varioskan microplate reader (Thermo Fisher Scientific, Waltham, MA, USA). The concentrations were calculated by means of a calibration curve that was created using IL-8 standards supplied with the kit.

### 4.15. Statistical Analysis

The experiments were performed three independent times in triplicates. Numeric data collected in the experiments were tested for normality with the Kolmogorov–Smirnov test. Multiple group comparisons were performed by means of ANOVA with post hoc Tukey’s test. Statistical tests were carried out and visualized with GraphPad Prism (GraphPad Software Inc., San Diego, CA, USA).

## Figures and Tables

**Table 1:** Concentrations of cytokines, chemokines and growth factors in conditioned media of MSCs of various origins cultured on plastic and dECM within 48 h. Concentrations were measured with use of a multiplex bead-based immunoassay. Data are presented as means ± SD (n = 2).

Analyte	pg/mL							
	MESCs		Fet-MSCs		WJ-MSCs		DP-MSCs	
	Plastic	dECM	Plastic	dECM	Plastic	dECM	Plastic	dECM
sCD40L	13.53 ± 0	21.84 ± 2.45	5.17 ± 0	17.58 ± 1.18	25.355 ± 2.5	39.94 ± 2.6	10.49 ± 0	18.43 ± 2.37
EGF	5.97 ± 0	7.195 ± 0.41	5.95 ± 0.92	5.97 ± 0	5.72 ± 0.11	8.91 ± 1.01	5.81 ± 1.96	5.885 ± 0.34
Eotaxin	4.11 ± 0	3.56 ± 0	2.99 ± 0	4.37 ± 0.36	44.785 ± 1.57	9.31 ± 0	5.61 ± 0	4.63 ± 0
FGF-2	<13.82	157.425 ± 10.81	<13.82	131.49 ± 2.79	23.72 ± 0	672.86 ± 1.2	31.92 ± 5.06	32.71 ± 0
FLT-3L	2.34 ± 0	2.5 ± 0	<0.91	1.14 ± 0.03	<0.91	1.73 ± 0.02	<0.91	<0.91
Fractalkine	<19.38	< 19.38	<19.38	<19.38	<19.38	<19.38	<19.38	<19.38
G-CSF	<3.39	374.21 ± 33.68	5.21 ± 0	843.07 ± 712.65	350.37 ± 23.83	2055.5 ± 543.76	35.58 ± 2.02	37.62 ± 21.99
GM-CSF	143.03 ± 0	917.01 ± 134.32	10.03 ± 9.82	4.915 ± 1.87	21.31 ± 2.88	236.49 ± 45.39	11.105 ± 9.11	9.525 ± 2.25
GRO-α	0.82 ± 0	179.2 ± 20.05	2.86 ± 1.46	2719.5 ± 392.44	2517 ± 214.96	9181.5 ± 651.24	8.04 ± 2.17	94.685 ± 3.95
HGF	<9.26	406.13 ± 0	<9.26	131.98 ± 0	43.18 ± 0	76.64 ± 0	13.15 ± 0	18.15 ± 0
IFNα2	<4.86	<4.86	<4.86	<4.86	<4.86	<4.86	<4.86	<4.86
IFNγ	<1.21	<1.21	<1.21	<1.21	<1.21	1.51 ± 0	<1.21	<1.21
IL-1a	5.89 ± 0	13.295 ± 3.93	<3.83	5.23 ± 0	47.355 ± 0.27	110.53 ± 5.19	<3.83	<3.83
IL-1b	<1.13	1.49 ± 0.12	<1.13	1.32 ± 0.12	3.88 ± 0.07	15.45 ± 1.64	<1.13	1.495 ± 0.12
IL-1RA	<1.46	<1.46	<1.46	<1.46	<1.46	<1.46	6.39 ± 0	<1.46
IL-2	<0.46	1.12 ± 0.25	<0.46	1.06 ± 0	0.73 ± 0.08	1.415 ± 0.16	<0.46	0.59 ± 0.05
IL-3	<0.79	0.95 ± 0	0.9 ± 0	<0.79	<0.79	<0.79	1.74 ± 0	<0.79
IL-4	<0.58	<0.58	<0.58	<0.58	<0.58	0.74 ± 0.07	1.04 ± 0.24	2.565 ± 0.79
IL-5	<0.24	<0.24	<0.24	<0.24	<0.24	<0.24	<0.24	<0.24
IL-6	40.52 ± 0	386.6 ± 39.31	12.87 ± 6.02	200.57 ± 10.67	4962 ± 217.78	5913.5 ± 64.34	683.64 ± 179.97	1131.5 ± 51.61
IL-7	1.39 ± 0	2.95 ± 0.74	1.67 ± 0.96	0.87 ± 0.16	< 0.60	1.19 ± 0.32	1.68 ± 0.82	0.96 ± 0.03
IL-8	26.47 ± 0	8112 ± 108.89	20.01 ± 9.78	6800.5 ± 509.82	3520 ± 28.28	6862 ± 657.60	117.41 ± 26.65	3008 ± 166.87
IL-9	0.92 ± 0	1.84 ± 0	0.805 ± 0.16	1.61 ± 0.32	2.005 ± 0.07	3.11 ± 0.28	1.495 ± 0.16	1.61 ± 0
IL-10	<0.89	<0.89	<0.89	<0.89	<0.89	0.99 ± 0.12	<0.89	<0.89
IL-12(p40)	4.46 ± 0	8.25 ± 1.16	4.46 ± 0	9.08 ± 0	19.54 ± 1.45	29.18 ± 0	6.65 ± 1.09	9.95 ± 1.23
IL-12(p70)	<2.24	<2.24	<2.24	<2.24	<2.24	<2.24	<2.24	<2.24
IL-13	<4.23	<4.23	<4.23	<4.23	<4.23	5.15 ± 0	<4.23	6.53 ± 0.28
IL-15	50.59 ± 0	43.88 ± 1.99	<2.45	4.68 ± 0.43	4.575 ± 0.28	10.035 ± 0.60	3.57 ± 0	2.97 ± 0.27
IL-17A	<1.11	1.6 ± 0	<1.11	1.32 ± 0.12	1.74 ± 0.19	4.02 ± 0.29	1.41 ± 0	1.23 ± 0
IL-17/IL-25	<19.59	<19.59	<19.59	<19.59	<19.59	<19.59	<19.59	<19.59
IL-17F	<16.95	<16.95	<16.95	<16.95	<16.95	<16.95	<16.95	<16.95
IL-18	<0.53	<0.53	<0.53	<0.53	<0.53	<0.53	<0.53	<0.53
IL-22	28.25 ± 0	32.725 ± 1.23	35.39 ± 9.23	33.55 ± 4.09	31.25 ± 0.84	55.05 ± 5.46	48.32 ± 13.65	57.48 ± 3.36
IL-27	<10.65	17.65 ± 4.19	11.98 ± 0	15.94 ± 1.76	15.94 ± 1.76	24.72 ± 1.37	14.58 ± 3.68	19.44 ± 3.18
IP-10	<2.36	<2.36	<2.36	<2.36	<2.36	<2.36	<2.36	<2.36
MCP-1	4547 ± 0	7390.5 ± 212.83	320.645 ± 59.30	5872 ± 548.71	8974 ± 87.68	12.274.5 ± 596.09	2336.5 ± 581.94	2218.5 ± 314.66
MCP-3	<6.94	37.47 ± 1.4	<6.94	62.67 ± 29.89	89.55 ± 3.84	4208.5 ± 3365.12	13.11 ± 0.39	17.78 ± 3.73
M-CSF	168.42 ± 0	163.5 ± 1.73	130.105 ± 12.29	150.24 ± 4.80	93.89 ± 0	104.55 ± 2.65	275.18 ± 23.17	214.97 ± 0
MDC	<0.62	<0.62	<0.62	<0.62	<0.62	<0.62	<0.62	<0.62
MIG	<5.47	<5.47	<5.47	<5.47	<5.47	<5.47	<5.47	<5.47
MIP-1a	10.71 ± 0	14.76 ± 0.25	8.27 ± 0.98	11.125 ± 0.58	13.66 ± 0.79	27.16 ± 0.50	11.31 ± 1.44	11.12 ± 0.58
MIP-1b	<0.42	<0.42	<0.42	<0.42	<0.42	0.76 ± 0.28	<0.42	<0.42
PDGF-AA	10.4 ± 0	<7.74	<7.74	<7.74	25.07 ± 0.12	121.03 ± 5.58	<7.74	<7.74
PDGF-AB/BB	15.23 ± 0	24.78 ± 1.89	12.46 ± 0	16.61 ± 1.95	17.99 ± 0	26.10 ± 3.76	24.06 ± 6.65	28.75 ± 3.72
RANTES	<1.15	27.31 ± 0.28	<1.15	9.4 ± 0.16	<1.15	1.62 ± 0	1.51 ± 0	9.47 ± 0.38
TGFα	<1.04	1.45 ± 0	<1.04	1.33 ± 0	1.21 ± 0	2.715 ± 0.36	1.21 ± 0	1.21 ± 0
TNFα	<4.02	4.28 ± 90	<4.02	<4.02	20.92 ± 1.34	28.405 ± 0.96	6.95 ± 1.92	12.125 ± 0.98
TNFβ	1.64 ± 0	1.47 ± 0	<1.38	1.98 ± 0	6.835 ± 0.16	9.455 ± 0.92	4.535 ± 1.19	11.81 ± 0.16
VEGF-A	82.38 ± 0	<2.36	39.08 ± 11.32	68.985 ± 1.54	<2.36	<2.36	262.215 ± 52.37	1040 ± 11.31

## Data Availability

The data that support the findings of this study are available from the corresponding author upon reasonable request.

## Supplementary Materials

The supporting information can be downloaded at: https://www.mdpi.com/article/10.3390/ijms25042419/s1.
