# Supplementary material for: The Decellularized Cell-Derived Extracellular Matrix Enhances the Paracrine Function of Human Mesenchymal Stromal/Stem Cells
Source: Int J Mol Sci. 2024 Feb 19;25(4):2419. doi: 10.3390/ijms25042419 (PMC10889327; doi:10.3390/ijms25042419)
Supplement: Supplementary file 1 [file ijms-25-02419-s001.zip › ijms-2857597-supplementary.pdf]

Supplementary Information

Figure S1. Scanning electron microscopy (SEM) images of extracellular matrix prior to decellularization (MSCs) and after (dECM Triton and dECM CHAPS according to decellularization method, respectively).

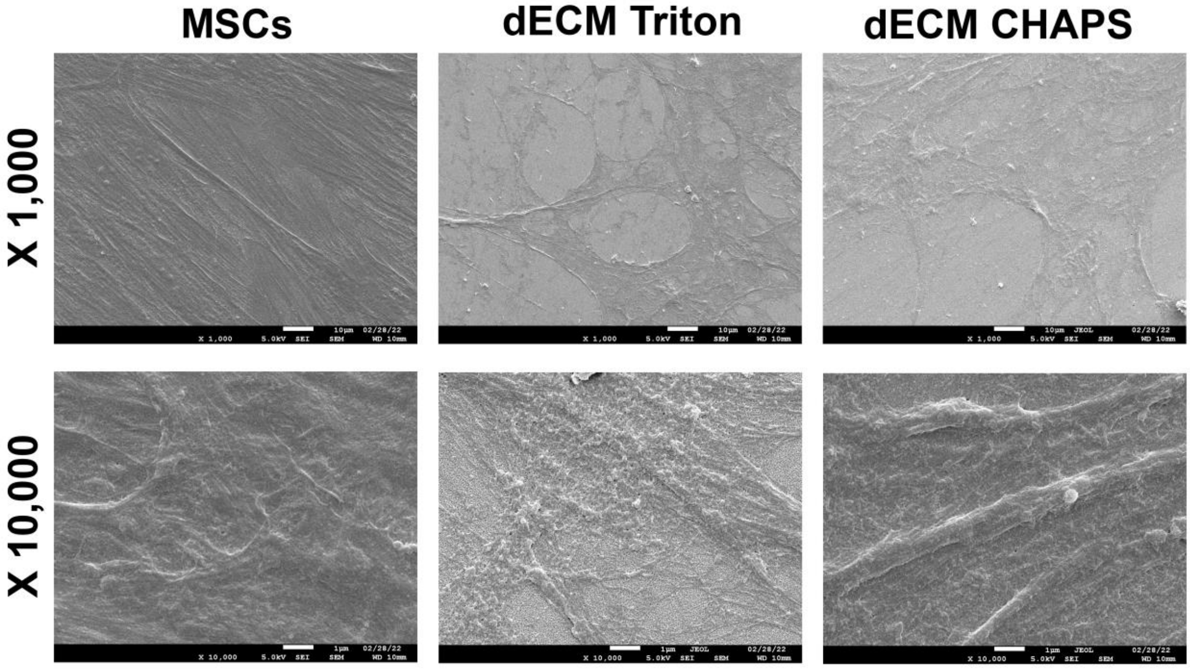

Figure S2. RT-qPCR heatmap depicts differential expression (dECM culture vs plastic culture) of paracrine factors mRNA in MESCs cultured on dECMs produced by MSCs of various origins. Values are presented as means (n=3) of binary logarithms of mRNA expression fold change. GAPDH was used as a reference gene. MESCs - human endometrium-derived MSCs, Fet-MSCs - human fetal bone marrow-derived MSCs, WJ-MSCs - Wharton jelly's-derived MSCs.

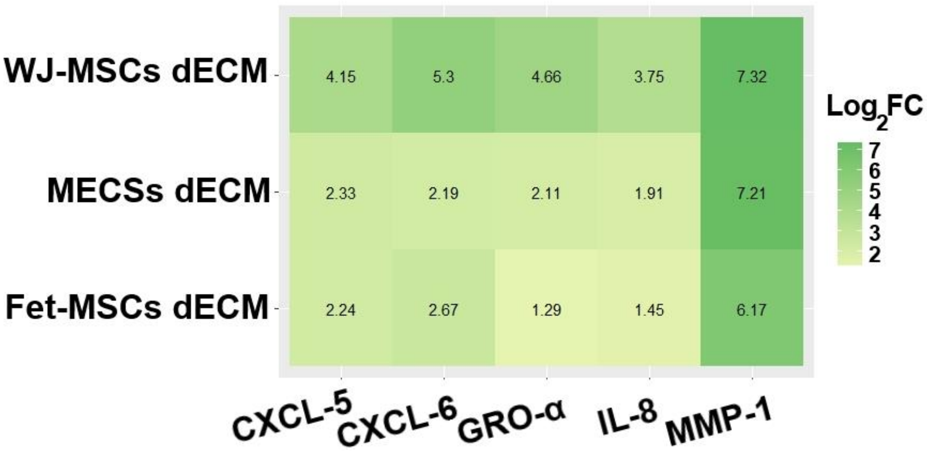

Figure S3. Differential expression (dECM culture vs plastic culture) of paracrine factors mRNA in MSCs cultured on dECMs (based on data published by Ragelle et al., 2017, GSE94667 Values are presented as means (n=3) of binary logarithms of mRNA expression fold change. AD – human adipose tissue-derived MSCs, BM – human bone marrow-derived MSCs, NHDF – human neonatal dermal fibroblasts. AD-ADdECM: AD cultured on dECM produced by AD etc.

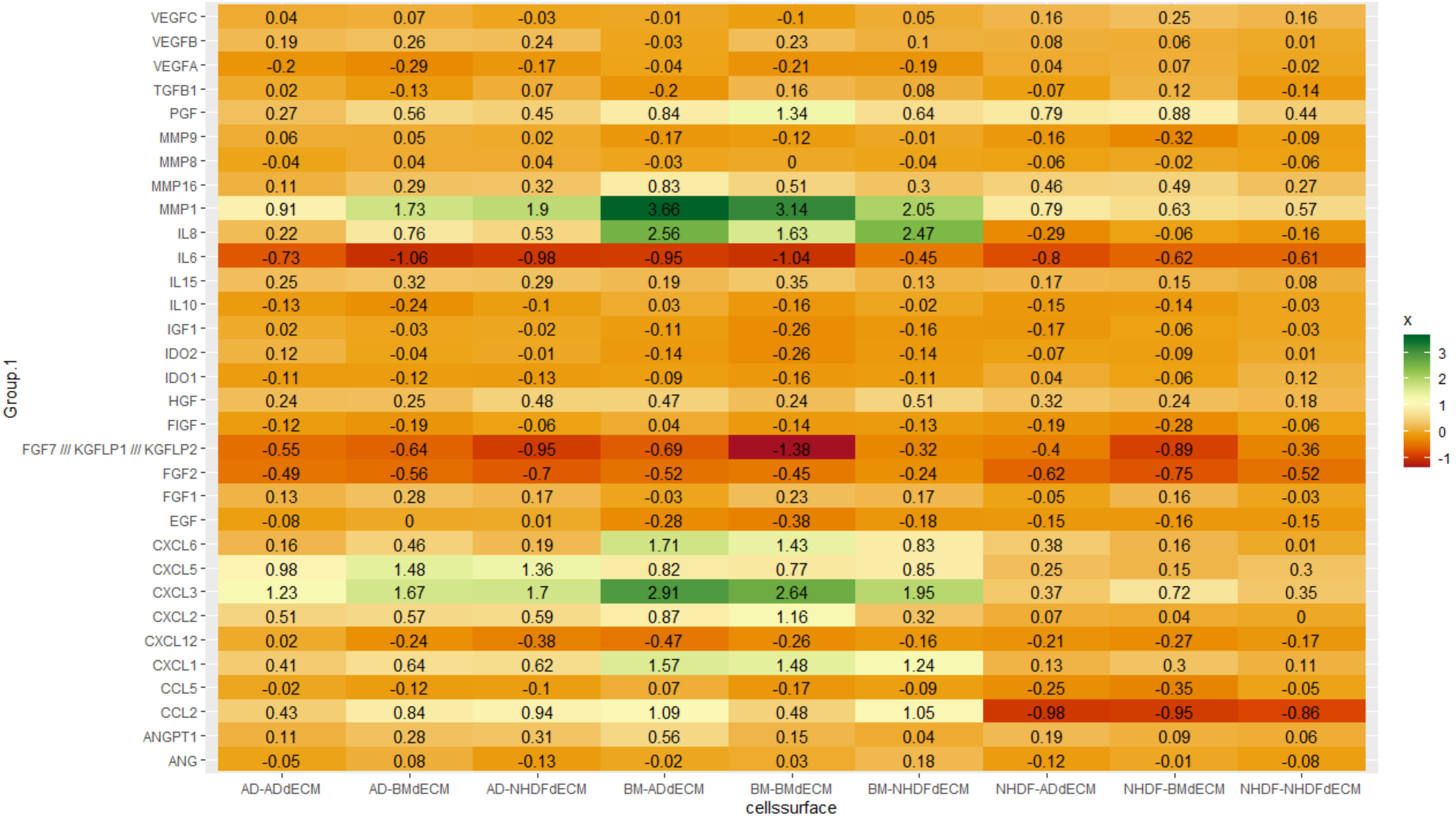

Figure S4. Differential expression (MSCs vs NHDF) of integrin subunits mRNA (based on data published by Ragelle et al., 2017, GSE94667) Values are presented as means (n=3) of binary logarithms of mRNA expression fold change.

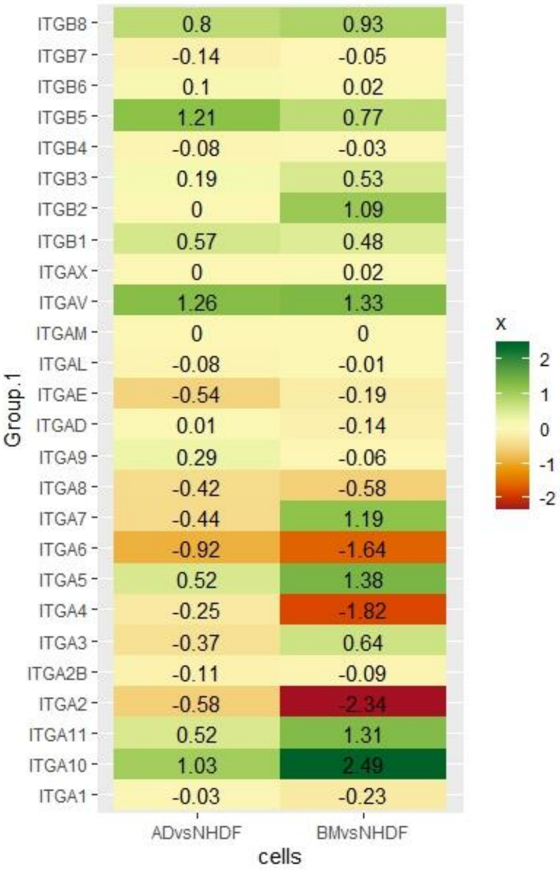

Table S1. Relative protein concentration in conditioned media measured with use of Bradford assay. Protein concentration in conditioned media correlates with number of cells. About 2.5-fold and 2-fold increase in protein concentration in conditioned media of MESCs and Fet-MSCs grown on dECM compared to plastic indicates enhanced proliferation. In contrast, DP-MSCs and WJ-MSCs grown on dECM do not demonstrate significant increase in protein concentration. OD_{595} – optical density measured on 595 nm.

	OD_{595}		
	CM-plastic	CM-dECM	dECM/plastic fold change
MESCs	0.127	0.308	2.42
Fet-MSCs	0.097	0.202	2.08
DP-MSCs	0.151	0.206	1.36
WJ-MSCs	0.442	0.523	1.20
